# Application of statistical procedures in analytical instrument testing

**DOI:** 10.1155/S1463924685000177

**Published:** 1985

**Authors:** W. Bablok, H. Passing

**Affiliations:** 1 Boehringer Mannheim GmbH Sandhofer Strasse 116 Mannheim 31 6800 Germany; 2 Hoechst A G Frankfurt/ Main Germany


Journal of Automatic Chemistry/*Journal of Clinical Laboratory Automation*, Vol. 7, No. 2 (Apr–Jun 1985), pp. 74–79

### Application of statistical procedures in analytical instrument testing

**W. Bablok**

*Boehringer Mannheim GmbH, Sandhofer Strasse 116, 6800 Mannheim 31, FR Germany*

**and H. Passing**

*Hoechst AG, Frankfurt/Main, FR Germany*

Evaluation of analytical instruments in clinical laboratories should be carried out in a systematic and uniform manner in order to provide data which can be compared and reproduced by different users, manufacturers and officials. Concepts for such an evaluation of instruments with continuous measurement values are presented in the ECCLS guidelines [1]. To quantify the results of an investigation, the data has to be condensed by the use of statistical methods. Obviously the design of an experiment and the statistical model used for evaluating the data are closely related. Only if the distributional structure of the experimental data is understood will it be possible to obtain an unbiased interpretation of the statistical results.

The first part of this paper covers all those properties of a new instrument which can be described by relatively simple statistical or numerical parameters: precision, analytical range limit and carry-over. Drift effects are usually assessed by visual inspection of a control chart. Method comparison studies require statistical procedures which allow hypothesis testing. Some researchers also make use of statistical tests for detecting deviant measurement values (outliers) which may be caused by interferences. These topics are dealt with in the second part of the paper.

### Descriptive statistics

*Investigation of precision*

The precision of an instrument in respect of a given method is a measure of the reproducibility of values within a fixed experimental design. It is described by the range in which repeated measurements scatter. Several statistical parameters can be used for the characterization of precision, each one leading to different regions of dispersion. The most common parameter is the standard deviation, which is the square root of the variance $s^2$:

$$s^2 = \sum_{i=1}^{n} \frac{(x_i - \bar{x})^2}{n-1}$$

where $n$ is the number of measurements in the series, $x_i$ is the $i$-th measurement in the series and $\bar{x}$ is the arithmetic mean of the series.

A derived quantity of $s$ is the coefficient of variation:

$$\mathrm{CV} = \frac{s}{\bar{x}} \cdot 100\,.$$

The formula above gives an unbiased estimation when all values $x_i$ have the same expected value $\mu$ and the same variance $\sigma^2$, and when the values are statistically independent. As a consequence, the series of measurements must be free of drift and erroneous values (outliers).

On the basis of $s$, a range around the mean can be defined in which a predefined percentage of values will be contained. For normally distributed data the regions $\bar{x} \pm 1 \cdot s$, $\bar{x} \pm 2 \cdot s$ and $\bar{x} \pm 3 \cdot s$ include about 68%, 95% and 99%. For an arbitrary distribution Tschebyscheff's inequality can be used to state the probability of a measurement value lying inside or outside a predefined region. A robust statistical parameter for describing precision is the $p$%-median distance $ma_p$. For $p = 68\%$, for instance, at least 68% of the measurements are in the interval $med \pm ma_{68}$, where $med$ represents the median of the measurements in the series. In general, the interval $med \pm ma_p$ [2] contains at least $p$% of the sample. This parameter is useful when the data structure is not known; for samples with $n < 15$ its use is not recommended.

In most cases the standard deviation $s$ is used to characterize precision. However, a researcher should consider carefully the distribution of the data when he examines the results and defines regions of dispersion. The reliability of the results depends on the sample size $n$; we advise as a minimum $n = 20$.

When evaluating the precision of a new instrument, experiments are carried out to assess 'within-run' precision and 'between-day' precision. 'Within-run' precision is calculated from a sequence of analyses between recalibration periods.

The measurements of the run are inspected after graphical presentation on usual control charts for errors, which could be caused by drift or deviant values. 'Between-day' precision is determined by obtaining duplicate results from a control specimen on $n$ consecutive days. The second value of each day is used for the calculation of the precision statistic. The proposal of $n = 10$ [1] might not be sufficient for a reliable estimation.

*Analytical range limit and carry-over*

The experimental layout for recording the analytical range of a method (instrument) is described in the ECCLS guidelines [1]. The upper and lower limit of the range are numerical quantities which are usually obtained without using statistical parameters.

Carry-over is caused by transfer of material from one assay to another. Procedures to treat carry-over are also described in the ECCLS guidelines [1].

### Method comparison studies

One of the central issues in instrument testing is comparative studies using samples from patients – these studies are a basic component of testing for accuracy [1]. In the study, $N$ independent samples from a (patient) population are investigated using the method (instrument) to be evaluated and a comparison method (instrument). The aim of the biometrical evaluation of the data obtained from such a study is to make statistical inferences about the equality of the methods or instruments. Under the premise of a linear relationship between the two methods in the form of

$$Y = a + bX$$

the estimated values for $a$ and $b$ are tested against the null hypothesis $a = 0$ and $b = 1$. If the estimated values differ only by chance from 0 and 1 at a predefined significance level, then the methods are equal.

To date, a number of different regression procedures have been used for biometrical evaluation of data from method comparison studies. Each one has specific theoretical requirements on the data. It is obvious that the reliability of a procedure depends largely on how far the data can meet these requirements.

The procedures can be classified into two categories:

(1) Procedures which depend on the assumption of normal distribution of the data.

(2) Procedures without distributional assumptions.

It is possible to differentiate within each category between procedures with one and those with both variables with error terms.

### *Procedures which assume normal distribution*

*Only one variable with error term*

*Linear regression* [3 and 4]: this procedure presumes that the measurements of method $X$ are obtained without any error. They represent the fixed values of the independent

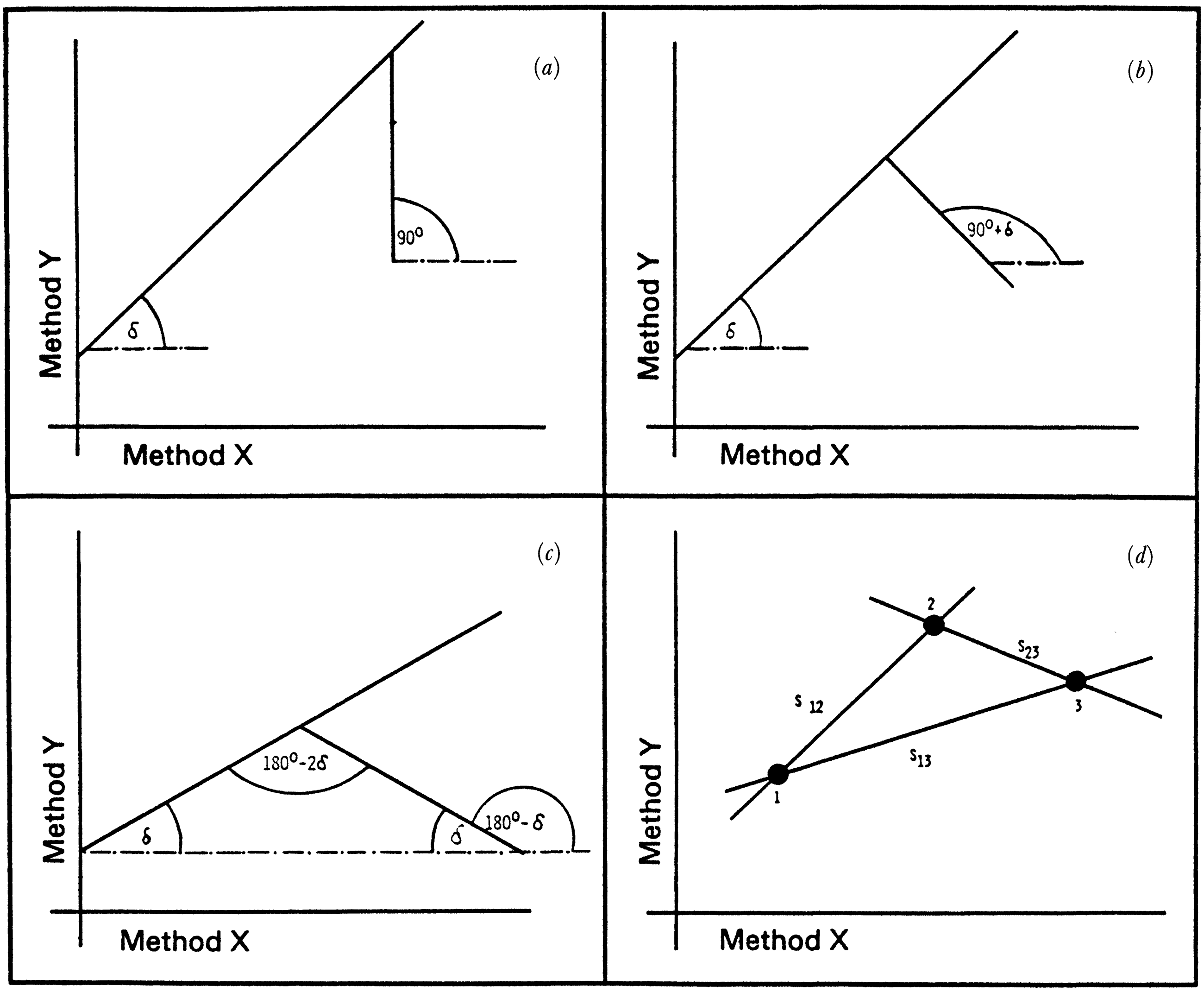

*Figure 1. Graphical presentation of regression lines. (a) Linear regression: slope of regression line: tg δ = b, slope of projection line: tg 90°. (b) Principal component (Deming procedure); slope of regression line: tg δ = b, slope of projection line: tg(90° + δ) = − 1/b. (c) Standardized principal component; slope of regression line: tg δ = b = $S_y/S_x$, slope of projection line: tg (180° − δ) = −b. (d) Theil, Bablok/Passing procedures: slope of the regression line: b = med($S_{ij}$).*

variable $x$. For the measurements of method $Y$ analytical errors are allowed, so that repeated measurements of one sample will scatter perpendicularly to the $x$-axis around their expected value on the regression line. The parameters $a$ and $b$ of the regression equation are determined by minimizing the sum of the squared distances between measurement points and regression line (see figure 1[*a*]). The method of least squares is sensitive to extreme data points, which may result in biased values of $a$ and $b$. A change in the assignment of the methods to the variables of the regression procedure results in new parameters which cannot be converted into the old ones by the regression equation. To obtain statistically unbiased results the procedure requires that:

(1) The measurements of the method assigned to the independent variable are not only free of error but also have fixed values.

(2) The measurements of the method assigned to the dependent variable have normally distributed error terms with constant variance over the concentration range.

(3) There is a linear relationship between the two methods.

*Both variables with error terms*

*Principal component analyses*: these regression procedures treat both methods alike; especially both methods may have measurements with analytical error terms. As a consequence the actual measurement point can deviate from the true value on the regression line in both the $x$- and $y$-direction. Therefore the calculation of the distance to the expected value has to be modified; two different situations can be considered:

(1) The standard deviation of the measurement errors is the same for both methods; then the distance is given by the vertical projection on the regression line, which is geometrically the shortest distance (see figure 1[*b*]). This procedure is known as the DEMING-procedure or orthogonal regression [5 and 6].

(2) The standard deviation of the measurement errors is different, but the ratio of the error variance to the total variance is equal for both methods. The distance is then given by the projection on the regression line which forms the angle $tg\delta = -S_y/S_x$ with the $x$-axis. ($S_x$ and $S_y$ are the standard deviation of the sampling distribution of method $X$ and $Y$.) The procedure is called standardized principal component or geometric mean regression [6 and 7] – the slope of the standardized principal component model is the geometric mean of the slopes of the two linear regression models (see figure 1[*c*]).

The slope of the projection line results in $-1/b$ for the DEMING-procedure and in $-b$ for the standardized principal component procedure. The parameters $a$ and $b$ of the regression line are calculated for both procedures by minimizing the sum of squared distances. However, for both procedures there are simple formulae for computing of $a$ and $b$ (see table 1).

*Table 1. Formulae for the computation of the parameters a and b of the regression procedures.*

| | Procedure | Estimation of $b$ | Estimation of $a$ |
|---|---|---|---|
| Univariate | Linear regression ($x$ independent) | $\frac{r \cdot S_y}{S_x}$ | $\bar{y} - b \cdot \bar{x}$ |
| | Linear regression ($y$ independent) | $\frac{S_y}{r \cdot S_x}$ | $\bar{y} - b \cdot \bar{x}$ |
| | Theil-regression ($x$ independent) | $med\,(S_{(i)});\ S_{ij} = \frac{y_i - y_j}{x_i - x_j}$ | Not proposed Pa/Ba can be used |
| Bivariate | Principal component (Deming-procedure) | $\frac{S^2_y - S^2_x + c_1}{2 \cdot r \cdot S_x \cdot S_y}$ | $\bar{y} - b \cdot \bar{x}$ |
| | Standardized principal component | $\frac{r}{\lvert r \rvert} \cdot \frac{S_y}{S_x}$ | $\bar{y} - b \cdot \bar{x}$ |
| | Passing/Bablok procedure | $med\,(S_{(i+K)});\ S_{ij} = \frac{y_i - y_j}{x_i - x_j}$ | $med\,(a_{(i)})$ $a_i = y_i - b\,x_i$ |

$$c_1 = +\sqrt{(S^2_y - S^2_x)^2 + 4r^2 \cdot S^2_x \cdot S^2_y};\quad r = \frac{S_{xy}}{S_x \cdot S_y}.$$

Extreme data points have a strong influence on the values of $a$ and $b$ and can lead to biased estimates. A change in the assignment of the methods to the variables does not alter the results of the method comparison.

To obtain statistically unbiased result the procedures require that:

(1) The measured values of the analyte are a random sample from a bivariate normal population.

(2) The analytical error terms are normally distributed and their variance is independent of concentration.

(3) There is a linear relationship between the two methods.

*Procedures without distributional assumptions*

*Only one variable with error term*

*THEIL procedure* [8]: again the independent variable $x$ is represented by fixed values. Each connecting line between two of the $N$ data points can be interpreted as an estimate of the slope of the regression line. Identical points and points with the same $x$-value do not contribute to the calculation of the regression line and are ignored; consequently at most $N \cdot (N-1)/2$ connecting lines can be calculated (see figure 1[*d*]). For a given pair of data points $(x_i, y_i)$ and $(x_j, y_j)$ the slope of the connecting line is given by:

$$s_{ij} = \frac{y_i - y_j}{x_i - x_j} \quad \text{for } 1 \le i < j \le N.$$

After ranking the slopes in ascending order:

$$s_{(1)} \le s_{(2)} \le s_{(3)} \le \cdots \le s_{(n)}$$

the slope of the regression line is obtained as the median of the ordered $s_{(i)}$. For the calculation of the intercept several proposals are discussed in the literature [9], each one depending on the previously determined value of $b$. A

change in the assignment of the methods to the variables can lead to different results of the method comparison.

To obtain statistically unbiased results the procedure requires that:

(1) The method asigned to the independent variable has measurements which are not only free of error but have fixed values.

(2) The method assigned to the dependent variable has continuously distributed measurements with constant error terms over the concentration range.

(3) There is a linear relationship between the two methods.

*Both variables with error terms*

*Passing/Bablok procedure* [10 and 11]: like the Theil procedure all possible connection lines between two data points are considered and their slopes calculated. If for two data points the $x$-values are equal (i.e. $x_i = x_j$), then the slope $s_{ij}$ is set to $\pm \infty$, where the sign is determined by the difference $y_i - y_j$. Again the slopes $s_{ij}$ are ranked in ascending order and the number of slopes with a value less than $-1$ is set to $K$. The parameter $b$ of the regression line is estimated by the slope which lies $K$ positions higher than the median of the $s_{(i)}$. For $K = 0$, i.e. $s_{(1)} > -1$, $b$ is represented directly by the median. The correction value $K$ ascertains that the assignment of the methods to the variables can be changed without altering the result of the method comparison.

To estimate the parameter $a$ of the regression line the values:

$$a_i = y_i - bx_i$$

are calculated for all $N$ data points. The intercept is then represented by the median of the sorted $a_i$:

$$a = med\ \{y_i - bx_i\} \text{ (see table 1).}$$

To obtain statistically unbiased results the procedure requires that:

(1) Both methods have continuously distributed measurements.

(2) There is a linear relationship between the two methods.

*Examination of linearity*

Since all regression procedures require a linear relationship between the two methods, a judgement of the parameters $a$ and $b$ is only meaningful after establishing the linearity of the data. In general, the question of a linear relationship is examined by visual inspection of the scatter plot. A more objective approach by means of a statistical test is desirable. Passing and Bablok [10] discuss two ways of testing linearity. In both cases scores with the values $+1$ or $-1$ are considered; these are derived from the data points and their relative location to the regression line. First the use of a run test is proposed to examine the randomness of the distribution of scores along the regression line $y = a + bx$. The second solution is based on the cusum concept, where an excess number of positive or negative scores along the regression line is tested by means of the Kolmogorov-Smirnov statistic. A judgement of linearity, of course, depends on the number of samples and the sampling distribution.

*Testing the equality*

After the parameters $a$ and $b$ of the regression line have been calculated, a researcher may wish to test whether or not the methods differ from each other. If the methods are equal then there is only a chance difference between $a$ and 0 and between $b$ and 1.

As in any statistical test a researcher has carefully to consider the choice of the number $N$ of samples to find a significant result for a relevant difference.

The predefined significance level is part of the statistical test, the size of a relevant difference is specific to the problem under study and has to be judged by the researcher. Obviously the main concern is in testing the parameter $b$. For each of the five regression procedures there is a statistical test provided the assumptions regarding the data are valid. For the preocedures with distributional assumptions the test for $b = 1$ makes use of the $t$-distribution, for the other procedures the test is related to the Kendall's $\tau$ statistic.

A test of the parameter $a$ is only proposed for the linear regression and the Passing and Bablok procedure.

Sometimes a researcher uses the matched pairs $t$-test or Wilcoxon test in method comparison studies for judging equality. However, with these tests only a difference in the means can be detected. The ECCLS guidelines [1] suggest their application to test $a = 0$ when $a$ is estimated by the standardized principal component procedure. Details on equality testing have been published by several authors [3, 6, 10 and 11].

*Discussion of the regression procedures*

The procedures described in the sections on 'Procedures which assume normal distribution' and 'Procedures without distributional assumptions' give reliable results as long as the data satisfy the requirements. A close look at the data structure of a standard comparison experiment shows that:

(1) Both methods have analytical error terms.

(2) The distribution of the error terms is frequently not normal.

(3) The variance of the error terms is seldom constant.

(4) The sampling distribution is mostly skew.

(5) Samples with considerable methodical differences can be present.

The way a procedure responds to data which violate its assumptions cannot be judged by an evaluation of experimental data. However, the behaviour of procedure has been investigated by Passing and Bablok [11] in a simulation study where well-defined data structures were used to estimate parameter $b$. All relevant combinations of the following conditions were considered:

(*a*) Relative size of measurement range.
(*b*) Sampling distribution (uniform or skew).
(*c*) Sample size.
(*d*) Precision of the methods.
(*e*) Distribution of the analytical error terms.
(*f*) Number and location of extreme data points.

The study showed that no procedure can be applied to method comparison experiments without restrictions. The following recommendations are suggested as a result of the study.

*Linear regression* procedures should not be used for biometrical evaluation, because the results are wrong in the majority of cases.

The *Theil* and *Deming* procedures are only of limited value when compared with the *standardized principal component* procedure. This procedure is reliable and the parameters *a* and *b* are easily computed. However, if the sampling distribution is skew or if the CVs are not constant or if extreme data points are present then biased estimation of *a* and *b* leading to false inferences about the equality of the methods must be expected. The procedure of *Passing/Bablok* shows reliable results in all of these situations; only when one of the CVs is higher than 7% and both CVs differ by a factor of two or more an *r*-fold determination with the less precise method is recommended. Because of the robustness of this procedure the problem of including or excluding extreme data points (outliers) does not arise. However, a separate investigation of outlying data points is advisable to locate method-specific differences. Usually this examination is carried out using a graphical presentation of the data. Samples which produced deviant values should be analysed again by both methods. Any measurement value should only be termed as an outlier

*Table 2. Sample size N for a uniform sampling distribution (significance level 5%). For a skew sampling distribution the size N should be multiplied by two.*

| | | | $b_{rel}$ <br> $1/b_{rel}$ | | | | | | | |
|---|---|---|---|---|---|---|---|---|---|---|
| Range *c* | $CV_x$ | $CV_y$ | 1·02<br>0·98 | 1·04<br>0·96 | 1·06<br>0·94 | 1·08<br>0·93 | 1·10<br>0·91 | 1·12<br>0·89 | 1·15<br>0·87 | 1·20<br>0·83 |
| | 2 | 2 | 60 | — | — | — | — | — | — | — |
| ∞ | 2 | 5 | + | 60 | 30 | — | — | — | — | — |
| | 5 | 2 | + | 60 | 30 | — | — | — | — | — |
| | 5 | 5 | + | 90 | 40 | 30 | — | — | — | — |
| | 5 | 7 | + | + | 70 | 40 | 30 | — | — | — |
| | 7 | 5 | + | + | 70 | 40 | 30 | — | — | — |
| | 7 | 7 | + | + | 80 | 50 | 30 | — | — | — |
| | 7 | 10 | + | + | + | 90 | 55 | 40 | 30 | — |
| | 10 | 7 | + | + | + | 90 | 55 | 40 | 30 | — |
| | 10 | 10 | + | + | + | 90 | 60 | 45 | 30 | — |
| | 10 | 13 | + | + | + | + | 90 | 70 | 45 | — |
| | 13 | 10 | + | + | + | + | 90 | 70 | 45 | — |
| | 13 | 13 | + | + | + | + | 90 | 75 | 50 | 30 |
| 4 | 2 | 2 | + | 45 | — | — | — | — | — | — |
| | 2 | 5 | + | + | 80 | 45 | 30 | — | — | — |
| | 5 | 2 | + | + | 80 | 45 | 30 | — | — | — |
| | 5 | 5 | + | + | + | 65 | 45 | — | — | — |
| | 5 | 7 | + | + | + | + | 70 | 45 | 35 | — |
| | 7 | 5 | + | + | + | + | 70 | 45 | 35 | — |
| | 7 | 7 | + | + | + | + | 75 | 55 | 40 | — |
| | 7 | 10 | + | + | + | + | + | + | 70 | 40 |
| | 10 | 7 | + | + | + | + | + | + | 70 | 40 |
| | 10 | 10 | + | + | + | + | + | + | 70 | 40 |
| | 10 | 13 | + | + | + | + | + | + | + | 70 |
| | 13 | 10 | + | + | + | + | + | + | + | 70 |
| | 13 | 13 | + | + | + | + | + | + | + | 70 |
| 2 | 2 | 2 | + | + | 60 | 35 | — | — | — | — |
| | 2 | 5 | + | + | + | + | + | 75 | 40 | — |
| | 5 | 2 | + | + | + | + | + | 75 | 40 | — |
| | 5 | 5 | + | + | + | + | + | 90 | 60 | 40 |
| | 5 | 7 | + | + | + | + | + | + | 90 | 60 |
| | 7 | 5 | + | + | + | + | + | + | 90 | 60 |
| | 7 | 7 | + | + | + | + | + | + | + | 65 |
| | 7 | 10 | + | + | + | + | + | + | + | + |
| | 10 | 7 | + | + | + | + | + | + | + | + |
| | 10 | 10 | + | + | + | + | + | + | + | + |

*The range c is an indicator for the ratio $c_{max}:c_{min}$ of the measurement values.*
*'+' indicates $N > 90$; '—' indicates $N < 30$, but we advise to have at least 30 samples.*

and be excluded from the data, if an analytical error was identified or the analyser declared the result as questionable. If the distribution of the data is known, a statistical test for detecting outliers can be used.

In addition, the study demonstrated that the size of the measurement range has a considerable influence on the outcome. The smaller the size of the range, the smaller the imprecision of the methods has to be.

For biometrical evaluation of method comparisons, the standardized principal component and the Passing/ Bablok procedures are advised. The first has its merits in the ease of computation, the second in reliability when testing equality.

*How to proceed in the evaluation*

To perform a method comparison study the following steps are suggested.

First, the precisions of the two methods (instruments) should be determined and the common concentration range is established. The question of what kind of sampling distribution can be obtained has to be considered and a value for $b_{rel}$ must be defined which indicates a relevant difference between the methods. From table 2, which is derived from the Passing and Bablok's results [11], the required sample size $N$ for the study is taken with respect to CVs, concentration range, $b_{rel}$ and sampling distribution. After the experimental data are available they are scrutinized in a scatter plot to detect and exclude gross measurement errors. Then the parameters $a$ and $b$ are estimated either by the Passing/ Bablok or standardized principal component procedure. To ensure a linear relationship between the data, linearity is tested as described by Passing and Bablok [10] or the data is visually inspected. If linearity is given, the test of the null hypothesis $a = 0$ and $b = 1$ is carried out [6 and 10]. If the null hypothesis is not rejected, then the equality of the methods is inferred. The experimental data and the results of the biometrical evaluation have to be documented.

In the final judgement of an instrument evaluation a researcher has to consider the outcome of the statistical calculations, as well as the findings which relate to his expertise in the field of clinical chemistry.

### References

1. *Guidelines for the Evaluation of Analysers in Clinical Chemistry*, 2nd draft, ECCLS Document Vol. 4, No. 1 (1984).
2. Eisenwiener, H. G., Bablok, W., Bardorff, W., Bender, R., Markowetz, D., Passing, H., Spaethe, R. and Specht, W., *Laboratoriums Medizin*, **7** (1983), 273.
3. Diem, K. and Lentner, C., *Wissenschaftliche Tabellen, 7. Auflage* (Ciba-Geigy AG, Basel, 1971), 175.
4. Richterich, R. and Colombo, J. P., *Klinische Chemie. Theorie, Praxis, Interpretation, 4. Auflage* (Karger, Basel, 1978), 30.
5. Deming, W. E., *Statistical Adjustment of Data* (John Wiley & Sons, New York, 1943), 1.
6. Feldmann, U., Schneider, B., Klinkers, H. and Haeckel, R., *Journal of Clinical Chemistry and Clinical Biochemistry,* **19** (1981), 121.
7. Averdunk, R. and Borner, K., *Journal of Clinical Chemistry and Clinical Biochemistry,* **8** (1970), 263.
8. Theil, H., *Proc. K. Ned. Akad. Wet.,* **Ser. A53** (1950), 386.
9. Maritz, J. S., *Distribution Free Statistical Methods* (Chapman and Hall, London, 1981), 265.
10. Passing, H. and Bablok, W., *Journal of Clinical Chemistry and Clinical Biochemistry,* **21** (1983), 709.
11. Passing, H. and Bablok, W., *Journal of Clinical Chemistry and Clinical Biochemistry,* **22** (1984), 431.

**BUDAPEST CHROMATOGRAPHY SYMPOSIUM**

*The 5th American-Eastern European Symposium on Liquid Chromatography: 11–14 June 1985*

To be held at the Research and Teaching Departments of the Semmelweis University Medical School, Nagyvárad tér 4, Budapest VIII the symposium will be conducted in English. The main topics at the meeting will be the analytical and preparative separation of biologically active compounds, the theory and practice of chromatography. Special sessions will be devoted to:

HPLC
HPTLC
Classical column liquid chromatography
Thin-layer chromatography
Forced flow (overpressured) thin-layer chromatography
Affinity chromatography
Electrophoresis techniques (PAGE, IEF, PAGIF, isotachophoresis)
Gas chromatography
Gas chromatography – mass spectrometry
Data processing in chromatography
Theoretical and practical aspects of separation of amines; amino acids, peptides, proteins; nucleic acids and their degradation products; drug, metabolites.

*More information (the registration fee is $100) from Congress Bureau Motesz, Budapest, PO Box 32, H 1361 Hungary*

